# Supplementary material for: Environment as a limiting factor of the historical global spread of mungbean
Source: eLife. 2023 May 19;12:e85725. doi: 10.7554/eLife.85725 (PMC10299821; doi:10.7554/eLife.85725)
Supplement: Supplementary file 1. — (a) Mungbean accessions from Vavilov Institute (VIR) collection. (b) Outgroup f3 statistics among all possible combinations of genetic group pairs. (c) Admixture f3 statistics among all possible population trios. (d) Mantel tests for isolation by distance of inferred genetic group (Q≥0.5). (e) Description of bioclimatic variables used in ecological niche modeling. (f) Pearson’s correlation coefficient between pairs of bioclimatic variables (denoted in lower triangle). (g) Comparison of bioclimatic variables among the four genetic groups analyzed with multivariate ANOVA (MANOVA). (h) Summary of ANOVA for bioclimatic variables. (i) Correlation between eight bioclimatic variables and climatic PC axes 1–4. (j) Comparison of summer growing season data including temperature and precipitation of May, July, and September among the four genetic groups analyzed with MANOVA. (k) ANOVA table for all evaluated field traits (phenology, reproduction, and size) as well as drought-related traits. (l) Mean of eight bioclimatic variables of the genetic groups [file elife-85725-supp1.docx]

Supplementary File 1

**Supplementary file 1a.** Mungbean accessions from Vavilov Institute (VIR) collection

| **Sample name** | **Country** | **Latitude** | **Longitude** | **NCBI SRA accession** | **NCBI Biosample accession** |
| --- | --- | --- | --- | --- | --- |
| 1.B | USA | NA | NA | SRR18125483 | SAMN26179197 |
| 100.A | Cyprus | 35.1856 | 33.3823 | SRR18125482 | SAMN26179198 |
| 101.A | Morocco | 33.9716 | -6.8498 | SRR18125266 | SAMN26179199 |
| 102.A | Israel | 32.4971 | 35.4973 | SRR18125200 | SAMN26179200 |
| 103.A | Indonesia | -6.5971 | 106.806 | SRR18125367 | SAMN26179201 |
| 104.B | Russia | 44.9003 | 131.8351 | SRR18125356 | SAMN26179202 |
| 105.A | Chile | -36.8305 | -73.1167 | SRR18125345 | SAMN26179203 |
| 106.A | India | 22.5587 | 88.2911 | SRR18125298 | SAMN26179204 |
| 107.A | India | 22.5726 | 88.3639 | SRR18125287 | SAMN26179205 |
| 108.A | India | 25.9821 | 85.6486 | SRR18125276 | SAMN26179206 |
| 109.A | Italy | 41.9028 | 12.4964 | SRR18125481 | SAMN26179207 |
| 10A | China | 47.1216 | 128.7382 | SRR18125398 | SAMN26179208 |
| 110.B | Portugal | 38.7223 | -9.1393 | SRR18125387 | SAMN26179209 |
| 111.A | Portugal | 38.7223 | -9.1393 | SRR18125376 | SAMN26179210 |
| 112.A | Russia | 45.0347 | 39.0978 | SRR18125329 | SAMN26179211 |
| 113.A | Tajikistan | 40.2675 | 69.6453 | SRR18125318 | SAMN26179212 |
| 114.B | India | 31.5204 | 74.3587 | SRR18125307 | SAMN26179213 |
| 115.B | India | 31.5204 | 74.3587 | SRR18125476 | SAMN26179214 |
| 116.A | Russia | 49.6152 | 127.9945 | SRR18125465 | SAMN26179215 |
| 116.B | Russia | 49.6152 | 127.9945 | SRR18125454 | SAMN26179216 |
| 117.B | Russia | 44.0281 | 131.3273 | SRR18125264 | SAMN26179217 |
| 118.A | Russia | 44.0118 | 131.3835 | SRR18125253 | SAMN26179218 |
| 119.A | Russia | 44.39 | 132.558 | SRR18125242 | SAMN26179219 |
| 11A | China | 47.1216 | 128.7382 | SRR18125231 | SAMN26179220 |
| 120.A | Argentina | -32.8895 | -68.8458 | SRR18125434 | SAMN26179221 |
| 122.A | Japan | 31.5969 | 130.5571 | SRR18125423 | SAMN26179222 |
| 123.B | Kyrgyzstan | 55.4649 | 65.3054 | SRR18125412 | SAMN26179223 |
| 124.B | Ethiopia | 12.9545 | 36.1573 | SRR18125223 | SAMN26179224 |
| 125.A | China | 42.5246 | 87.5396 | SRR18125212 | SAMN26179225 |
| 126.A | China | 42.5246 | 87.5396 | SRR18125201 | SAMN26179226 |
| 127.A | China | 42.5246 | 87.5396 | SRR18125199 | SAMN26179227 |
| 128.A | Democratic Republic of the Congo | 1.9293 | 30.0492 | SRR18125198 | SAMN26179228 |
| 128.B | Democratic Republic of the Congo | 1.9293 | 30.0492 | SRR18125197 | SAMN26179229 |
| 129.A | Korea | 37.5665 | 126.978 | SRR18125196 | SAMN26179230 |
| 12A | China | 47.1216 | 128.7382 | SRR18125195 | SAMN26179231 |
| 13.A | Iran | 32.4279 | 53.688 | SRR18125194 | SAMN26179232 |
| 130.A | Korea | 37.5665 | 126.978 | SRR18125371 | SAMN26179233 |
| 131.A | Korea | 37.5665 | 126.978 | SRR18125370 | SAMN26179234 |
| 132.B | Korea | 40.3399 | 127.5101 | SRR18125369 | SAMN26179235 |
| 133.A | Korea | 40.3399 | 127.5101 | SRR18125368 | SAMN26179236 |
| 134.A | Korea | 40.3399 | 127.5101 | SRR18125366 | SAMN26179237 |
| 135.A | Korea | 40.3399 | 127.5101 | SRR18125365 | SAMN26179238 |
| 136.A | Korea | 40.3399 | 127.5101 | SRR18125364 | SAMN26179239 |
| 137.A | China | 42.5246 | 87.5396 | SRR18125363 | SAMN26179240 |
| 138.A | China | 39.4677 | 75.9938 | SRR18125362 | SAMN26179241 |
| 139.A | Uzbekistan | 41.2995 | 69.2401 | SRR18125361 | SAMN26179242 |
| 14.B | Uzbekistan | 39.7681 | 64.4556 | SRR18125360 | SAMN26179243 |
| 140.B | China | 42.5246 | 87.5396 | SRR18125359 | SAMN26179244 |
| 141.A | China | 43.8256 | 87.6168 | SRR18125358 | SAMN26179245 |
| 142.B | China | 42.5246 | 87.5396 | SRR18125357 | SAMN26179246 |
| 144.A | Ukraine | 48.4647 | 35.0462 | SRR18125355 | SAMN26179247 |
| 145.A | Brazil | -22.9329 | -47.0738 | SRR18125354 | SAMN26179248 |
| 146.B | Kazakhstan | 43.222 | 76.8512 | SRR18125353 | SAMN26179249 |
| 147.A | Turkey | 39.7646 | 30.4559 | SRR18125352 | SAMN26179250 |
| 148.B | Turkey | 39.7646 | 30.4559 | SRR18125351 | SAMN26179251 |
| 149.A | Ukraine | 48.0386 | 30.9497 | SRR18125350 | SAMN26179252 |
| 151.A | Tajikistan | 38.5598 | 68.787 | SRR18125349 | SAMN26179253 |
| 154.A | Brazil | -14.235 | -51.9253 | SRR18125348 | SAMN26179254 |
| 155.A | United Kingdom | 52.3555 | -1.1743 | SRR18125347 | SAMN26179255 |
| 156.A | India | 25.9821 | 85.6486 | SRR18125346 | SAMN26179256 |
| 157.A | India | 25.9821 | 85.6486 | SRR18125344 | SAMN26179257 |
| 158.A | India | 25.9821 | 85.6486 | SRR18125343 | SAMN26179258 |
| 159.B | India | 25.9821 | 85.6486 | SRR18125342 | SAMN26179259 |
| 16.A | Uzbekistan | 39.7681 | 64.4556 | SRR18125341 | SAMN26179260 |
| 160.A | Uzbekistan | 40.8154 | 72.2837 | SRR18125340 | SAMN26179261 |
| 161.A | Uzbekistan | 40.8154 | 72.2837 | SRR18125339 | SAMN26179262 |
| 162.A | South Africa | -26.7145 | 27.097 | SRR18125338 | SAMN26179263 |
| 163.A | South Africa | -26.7145 | 27.097 | SRR18125337 | SAMN26179264 |
| 164.A | South Africa | -26.7145 | 27.097 | SRR18125336 | SAMN26179265 |
| 165.B | USA | 39.9526 | -75.1652 | SRR18125299 | SAMN26179266 |
| 166.A | USA | 39.9526 | -75.1652 | SRR18125297 | SAMN26179267 |
| 167.B | Kyrgyzstan | 42.8224 | 75.3179 | SRR18125296 | SAMN26179268 |
| 168.A | Kyrgyzstan | 42.8224 | 75.3179 | SRR18125295 | SAMN26179269 |
| 17.B | Uzbekistan | 39.7681 | 64.4556 | SRR18125294 | SAMN26179270 |
| 170.A | Russia | 43.2562 | 46.5893 | SRR18125293 | SAMN26179271 |
| 171.A | Russia | 43.2562 | 46.5893 | SRR18125292 | SAMN26179272 |
| 172.B | Iran | 32.4279 | 53.688 | SRR18125291 | SAMN26179273 |
| 173.A | Iran | 32.4279 | 53.688 | SRR18125290 | SAMN26179274 |
| 174.A | Korea | 40.3399 | 127.5101 | SRR18125289 | SAMN26179275 |
| 175.A | Uzbekistan | 40.2504 | 63.2032 | SRR18125288 | SAMN26179276 |
| 175.B | Uzbekistan | 40.2504 | 63.2032 | SRR18125286 | SAMN26179277 |
| 176.A | Uzbekistan | 40.2504 | 63.2032 | SRR18125285 | SAMN26179278 |
| 177.A | Uzbekistan | 40.2504 | 63.2032 | SRR18125284 | SAMN26179279 |
| 178.A | Uzbekistan | 40.2504 | 63.2032 | SRR18125283 | SAMN26179280 |
| 179.A | Uzbekistan | 39.9208 | 66.4271 | SRR18125282 | SAMN26179281 |
| 18.B | Uzbekistan | 39.7681 | 64.4556 | SRR18125281 | SAMN26179282 |
| 180.A | Uzbekistan | 39.9208 | 66.4271 | SRR18125280 | SAMN26179283 |
| 181.A | Uzbekistan | 39.9208 | 66.4271 | SRR18125279 | SAMN26179284 |
| 182.B | Uzbekistan | 39.9208 | 66.4271 | SRR18125278 | SAMN26179285 |
| 183.A | Uzbekistan | 40.3734 | 71.7978 | SRR18125277 | SAMN26179286 |
| 184.A | China | 41.482754 | 85.626702 | SRR18125275 | SAMN26179287 |
| 187.A | China | 38.10222 | 76.993816 | SRR18125274 | SAMN26179288 |
| 188.B | China | 41.1675 | 80.2634 | SRR18125273 | SAMN26179289 |
| 189.A | China | 41.1675 | 80.2634 | SRR18125272 | SAMN26179290 |
| 19.B | Iran | 32.4279 | 53.688 | SRR18125271 | SAMN26179291 |
| 190.A | China | 42.9513 | 89.1898 | SRR18125270 | SAMN26179292 |
| 191.A | China | 41.175324 | 85.660861 | SRR18125269 | SAMN26179293 |
| 192.A | India | 31.8183 | 75.2071 | SRR18125268 | SAMN26179294 |
| 193.A | India | 31.326 | 75.5762 | SRR18125267 | SAMN26179295 |
| 193.B | India | 31.326 | 75.5762 | SRR18125265 | SAMN26179296 |
| 194.A | India | 30.3752 | 76.7821 | SRR18125480 | SAMN26179297 |
| 195.A | India | 28.7041 | 77.1025 | SRR18125407 | SAMN26179298 |
| 195.B | India | 28.7041 | 77.1025 | SRR18125406 | SAMN26179299 |
| 197.A | India | 17.6599 | 75.9064 | SRR18125405 | SAMN26179300 |
| 199.A | Senegal | 14.4974 | -14.4524 | SRR18125404 | SAMN26179301 |
| 1A | USA | NA | NA | SRR18125403 | SAMN26179302 |
| 201.B | Pakistan | 31.5204 | 74.3587 | SRR18125402 | SAMN26179303 |
| 202.B | Canada | 43.6502 | -79.9036 | SRR18125401 | SAMN26179304 |
| 203.A | Ethiopia | 9.3126 | 42.1227 | SRR18125400 | SAMN26179305 |
| 204.B | Indonesia | -6.5971 | 106.806 | SRR18125399 | SAMN26179306 |
| 205.A | Indonesia | -6.5971 | 106.806 | SRR18125397 | SAMN26179307 |
| 205.B | Indonesia | -6.5971 | 106.806 | SRR18125396 | SAMN26179308 |
| 206.A | Hungary | 47.1625 | 19.5033 | SRR18125395 | SAMN26179309 |
| 207.A | Viet Nam | 14.0583 | 108.2772 | SRR18125394 | SAMN26179310 |
| 21.B | Kazakhstan | 51.1605 | 71.4704 | SRR18125393 | SAMN26179311 |
| 210.A | Afghanistan | 36.6926 | 67.118 | SRR18125392 | SAMN26179312 |
| 212.A | Viet Nam | 21.0278 | 105.8342 | SRR18125391 | SAMN26179313 |
| 215.A | Guinea | 8.5383 | -9.4728 | SRR18125390 | SAMN26179314 |
| 216.A | Uzbekistan | 39.972132 | 65.558096 | SRR18125389 | SAMN26179315 |
| 217.B | Uzbekistan | 40.162885 | 66.227209 | SRR18125388 | SAMN26179316 |
| 218.A | Uzbekistan | 40.013465 | 64.943243 | SRR18125386 | SAMN26179317 |
| 219.A | Turkmenistan | 40.243331 | 59.540314 | SRR18125385 | SAMN26179318 |
| 22.B | Kazakhstan | 42.3417 | 69.5901 | SRR18125384 | SAMN26179319 |
| 220.A | Turkmenistan | 40.243331 | 59.540314 | SRR18125383 | SAMN26179320 |
| 223.A | Uzbekistan | 40.483568 | 70.546311 | SRR18125382 | SAMN26179321 |
| 224.A | Iran | 32.6539 | 51.666 | SRR18125381 | SAMN26179322 |
| 225.A | Pakistan | 30.3753 | 69.3451 | SRR18125380 | SAMN26179323 |
| 226.B | Pakistan | 30.3753 | 69.3451 | SRR18125379 | SAMN26179324 |
| 227.A | Pakistan | 30.3753 | 69.3451 | SRR18125378 | SAMN26179325 |
| 228.B | Turkmenistan | 39.0041 | 63.5688 | SRR18125377 | SAMN26179326 |
| 229.A | Pakistan | 31.4504 | 73.135 | SRR18125375 | SAMN26179327 |
| 23.A | Kazakhstan | 42.3417 | 69.5901 | SRR18125374 | SAMN26179328 |
| 230.A | Pakistan | 31.4504 | 73.135 | SRR18125373 | SAMN26179329 |
| 230.B | Pakistan | 31.4504 | 73.135 | SRR18125372 | SAMN26179330 |
| 231.A | Tanzania | -6.369 | 34.8888 | SRR18125335 | SAMN26179331 |
| 232.A | Iran | 34.3277 | 47.0778 | SRR18125334 | SAMN26179332 |
| 233.A | Iran | 32.4279 | 53.688 | SRR18125333 | SAMN26179333 |
| 234.A | India | 28.6139 | 77.209 | SRR18125332 | SAMN26179334 |
| 235.A | Pakistan | 33.5651 | 73.0169 | SRR18125331 | SAMN26179335 |
| 235.B | Pakistan | 33.5651 | 73.0169 | SRR18125330 | SAMN26179336 |
| 236.A | Pakistan | 34.0155 | 71.6888 | SRR18125328 | SAMN26179337 |
| 237.A | Pakistan | 28.6001 | 77.227 | SRR18125327 | SAMN26179338 |
| 238.A | Egypt | 26.8206 | 30.8025 | SRR18125326 | SAMN26179339 |
| 239.A | Kazakhstan | 44.7689 | 77.5573 | SRR18125325 | SAMN26179340 |
| 24.A | Uzbekistan | 41.4065 | 60.3685 | SRR18125324 | SAMN26179341 |
| 240.A | Uzbekistan | 39.4065 | 67.1418 | SRR18125323 | SAMN26179342 |
| 240.B | Uzbekistan | 39.4065 | 67.1418 | SRR18125322 | SAMN26179343 |
| 241.A | Uzbekistan | 40.023044 | 67.433724 | SRR18125321 | SAMN26179344 |
| 242.A | Uzbekistan | 39.982851 | 67.486778 | SRR18125320 | SAMN26179345 |
| 244.A | Uzbekistan | 41.773406 | 63.780613 | SRR18125319 | SAMN26179346 |
| 245.A | Kazakhstan | 43.47491 | 75.335144 | SRR18125317 | SAMN26179347 |
| 246.A | Algeria | 35.6971 | -0.6308 | SRR18125316 | SAMN26179348 |
| 247.A | Tanzania | -5.0425 | 32.8197 | SRR18125315 | SAMN26179349 |
| 249.A | Australia | -32.9283 | 151.7817 | SRR18125314 | SAMN26179350 |
| 25.A | Uzbekistan | 41.4065 | 60.3685 | SRR18125313 | SAMN26179351 |
| 250.B | Russia | NA | NA | SRR18125312 | SAMN26179352 |
| 251.B | USA | NA | NA | SRR18125311 | SAMN26179353 |
| 252.A | Afghanistan | 34.7602 | 69.8121 | SRR18125310 | SAMN26179354 |
| 253.A | Afghanistan | 34.7602 | 69.8121 | SRR18125309 | SAMN26179355 |
| 254.A | Yemen | 15.5527 | 48.5164 | SRR18125308 | SAMN26179356 |
| 254.B | Yemen | 15.5527 | 48.5164 | SRR18125306 | SAMN26179357 |
| 255.A | Afghanistan | 36.6926 | 67.118 | SRR18125305 | SAMN26179358 |
| 256.A | Afghanistan | 36.6926 | 67.118 | SRR18125304 | SAMN26179359 |
| 257.B | Afghanistan | 36.6926 | 67.118 | SRR18125303 | SAMN26179360 |
| 258.B | Afghanistan | 33.9391 | 67.71 | SRR18125302 | SAMN26179361 |
| 259.B | Colombia | 4.5709 | -74.2973 | SRR18125301 | SAMN26179362 |
| 26.B | Uzbekistan | 41.4065 | 60.3685 | SRR18125300 | SAMN26179363 |
| 260.A | Philippines | 14.5995 | 120.9842 | SRR18125479 | SAMN26179364 |
| 261.B | Kenya | -1.2921 | 36.8219 | SRR18125478 | SAMN26179365 |
| 262.A | Kenya | -1.2921 | 36.8219 | SRR18125477 | SAMN26179366 |
| 263.A | Kenya | -1.2921 | 36.8219 | SRR18125475 | SAMN26179367 |
| 264.A | Kenya | -1.2921 | 36.8219 | SRR18125474 | SAMN26179368 |
| 265.B | Kenya | -1.2921 | 36.8219 | SRR18125473 | SAMN26179369 |
| 266.A | Kenya | -1.2921 | 36.8219 | SRR18125472 | SAMN26179370 |
| 267.A | Kenya | -1.2921 | 36.8219 | SRR18125471 | SAMN26179371 |
| 268.A | Kenya | -1.2921 | 36.8219 | SRR18125470 | SAMN26179372 |
| 269.B | Kenya | -1.2921 | 36.8219 | SRR18125469 | SAMN26179373 |
| 27.A | Uzbekistan | 39.7681 | 64.4556 | SRR18125468 | SAMN26179374 |
| 270.A | Kenya | -1.2921 | 36.8219 | SRR18125467 | SAMN26179375 |
| 271.A | South Korea | 35.8987 | 127.0392 | SRR18125466 | SAMN26179376 |
| 272.A | Australia | NA | NA | SRR18125464 | SAMN26179377 |
| 273.A | Kenya | -1.2921 | 36.8219 | SRR18125463 | SAMN26179378 |
| 274.A | Kenya | -1.2921 | 36.8219 | SRR18125462 | SAMN26179379 |
| 275.A | Kenya | -1.2921 | 36.8219 | SRR18125461 | SAMN26179380 |
| 276.A | Kenya | -1.2921 | 36.8219 | SRR18125460 | SAMN26179381 |
| 277.B | Kazakhstan | 43.1521 | 68.2581 | SRR18125459 | SAMN26179382 |
| 278.B | Kazakhstan | 43.0631 | 69.0851 | SRR18125458 | SAMN26179383 |
| 279.A | Kazakhstan | 41.5295 | 69.4133 | SRR18125457 | SAMN26179384 |
| 28.A | Russia | 42.1432 | 47.095 | SRR18125456 | SAMN26179385 |
| 280.B | Kazakhstan | 41.5295 | 69.4133 | SRR18125455 | SAMN26179386 |
| 281.B | South Korea | 35.8987 | 127.0392 | SRR18125453 | SAMN26179387 |
| 282.B | South Korea | 35.8987 | 127.0392 | SRR18125452 | SAMN26179388 |
| 283.B | South Korea | 35.8987 | 127.0392 | SRR18125451 | SAMN26179389 |
| 284.A | Tajikistan | 38.0116 | 71.003 | SRR18125450 | SAMN26179390 |
| 285.A | Tajikistan | 37.074793 | 67.957222 | SRR18125449 | SAMN26179391 |
| 286.A | Tajikistan | 37.028926 | 68.004059 | SRR18125448 | SAMN26179392 |
| 287.A | Tajikistan | 39.179338 | 68.012339 | SRR18125447 | SAMN26179393 |
| 288.A | Philippines | 14.5995 | 120.9842 | SRR18125446 | SAMN26179394 |
| 289.B | Philippines | 14.5995 | 120.9842 | SRR18125445 | SAMN26179395 |
| 290.A | China | 40.2374 | 116.2305 | SRR18125444 | SAMN26179396 |
| 290.B | China | 40.2374 | 116.2305 | SRR18125263 | SAMN26179397 |
| 291.A | China | 40.2374 | 116.2305 | SRR18125262 | SAMN26179398 |
| 292.A | China | 30.7378 | 112.2384 | SRR18125261 | SAMN26179399 |
| 293.A | China | 30.7378 | 112.2384 | SRR18125260 | SAMN26179400 |
| 294.A | China | 40.2374 | 116.2305 | SRR18125259 | SAMN26179401 |
| 295.B | China | 37.8957 | 114.9042 | SRR18125258 | SAMN26179402 |
| 296.A | China | 37.8957 | 114.9042 | SRR18125257 | SAMN26179403 |
| 297.A | China | 47.1216 | 128.7382 | SRR18125256 | SAMN26179404 |
| 298.B | China | 47.1216 | 128.7382 | SRR18125255 | SAMN26179405 |
| 299.A | China | 40.2374 | 116.2305 | SRR18125254 | SAMN26179406 |
| 2A | China | 47.1216 | 128.7382 | SRR18125252 | SAMN26179407 |
| 3.B | China | 47.1216 | 128.7382 | SRR18125251 | SAMN26179408 |
| 30.A | Russia | 42.1432 | 47.095 | SRR18125250 | SAMN26179409 |
| 300.A | China | 47.1216 | 128.7382 | SRR18125249 | SAMN26179410 |
| 31.A | Russia | 42.1432 | 47.095 | SRR18125248 | SAMN26179411 |
| 32.B | Russia | 42.1432 | 47.095 | SRR18125247 | SAMN26179412 |
| 33.A | USA | NA | NA | SRR18125246 | SAMN26179413 |
| 34.A | USA | NA | NA | SRR18125245 | SAMN26179414 |
| 34.B | USA | NA | NA | SRR18125244 | SAMN26179415 |
| 35.A | Russia | NA | NA | SRR18125243 | SAMN26179416 |
| 36.A | Russia | 43.1198 | 131.8869 | SRR18125241 | SAMN26179417 |
| 37.A | USA | NA | NA | SRR18125240 | SAMN26179418 |
| 38.B | China | 44.9188 | 130.5244 | SRR18125239 | SAMN26179419 |
| 39.A | Iran | 36.3394 | 59.4698 | SRR18125238 | SAMN26179420 |
| 3A | China | 47.1216 | 128.7382 | SRR18125237 | SAMN26179421 |
| 40.B | Iran | 36.3394 | 59.4698 | SRR18125236 | SAMN26179422 |
| 41.B | Iran | 32.4279 | 53.688 | SRR18125235 | SAMN26179423 |
| 42.A | Iran | 32.4279 | 53.688 | SRR18125234 | SAMN26179424 |
| 43.A | Iran | 32.4279 | 53.688 | SRR18125233 | SAMN26179425 |
| 44.B | Turkmenistan | 37.9153 | 58.0897 | SRR18125232 | SAMN26179426 |
| 45.A | Turkmenistan | 37.9153 | 58.0897 | SRR18125230 | SAMN26179427 |
| 46.A | Turkmenistan | 37.9153 | 58.0897 | SRR18125443 | SAMN26179428 |
| 47.A | Turkmenistan | 37.9153 | 58.0897 | SRR18125442 | SAMN26179429 |
| 48.B | Turkmenistan | 37.9153 | 58.0897 | SRR18125441 | SAMN26179430 |
| 49.B | Turkmenistan | 37.9172 | 58.0907 | SRR18125440 | SAMN26179431 |
| 4A | China | 47.1216 | 128.7382 | SRR18125439 | SAMN26179432 |
| 5.B | China | 47.1216 | 128.7382 | SRR18125438 | SAMN26179433 |
| 50.A | Turkmenistan | 37.9172 | 58.0907 | SRR18125437 | SAMN26179434 |
| 51.B | Turkmenistan | 37.9601 | 58.3261 | SRR18125436 | SAMN26179435 |
| 52.A | Turkmenistan | 37.9601 | 58.3261 | SRR18125435 | SAMN26179436 |
| 53.B | USA | 40.1605 | -103.2144 | SRR18125433 | SAMN26179437 |
| 54.A | USA | 40.1605 | -103.2144 | SRR18125432 | SAMN26179438 |
| 55.B | Ukraine | 48.3794 | 31.1656 | SRR18125431 | SAMN26179439 |
| 56.A | Kazakhstan | 43.3667 | 68.4094 | SRR18125430 | SAMN26179440 |
| 57.A | Iran | 34.7608 | 48.3988 | SRR18125429 | SAMN26179441 |
| 58.A | Iran | 35.6892 | 51.389 | SRR18125428 | SAMN26179442 |
| 59.B | Kazakhstan | 43.3667 | 68.4094 | SRR18125427 | SAMN26179443 |
| 6.B | China | 47.1216 | 128.7382 | SRR18125426 | SAMN26179444 |
| 60.A | Kazakhstan | 42.2663 | 68.1431 | SRR18125425 | SAMN26179445 |
| 61.A | Uzbekistan | 41.2995 | 69.2401 | SRR18125424 | SAMN26179446 |
| 62.A | Uzbekistan | 41.2995 | 69.2401 | SRR18125422 | SAMN26179447 |
| 63.A | Uzbekistan | 41.2995 | 69.2401 | SRR18125421 | SAMN26179448 |
| 64.B | Uzbekistan | 41.2995 | 69.2401 | SRR18125420 | SAMN26179449 |
| 65.B | Uzbekistan | 41.2995 | 69.2401 | SRR18125419 | SAMN26179450 |
| 66.B | Uzbekistan | 41.2995 | 69.2401 | SRR18125418 | SAMN26179451 |
| 67.A | Uzbekistan | 41.2995 | 69.2401 | SRR18125417 | SAMN26179452 |
| 68.A | Uzbekistan | 41.2995 | 69.2401 | SRR18125416 | SAMN26179453 |
| 69.A | Uzbekistan | 40.4915 | 68.7811 | SRR18125415 | SAMN26179454 |
| 70.A | Uzbekistan | 39.627 | 66.975 | SRR18125414 | SAMN26179455 |
| 71.A | Uzbekistan | 39.627 | 66.975 | SRR18125413 | SAMN26179456 |
| 72.A | Uzbekistan | 39.627 | 66.975 | SRR18125411 | SAMN26179457 |
| 73.B | Uzbekistan | 39.627 | 66.975 | SRR18125410 | SAMN26179458 |
| 74.A | Uzbekistan | 39.627 | 66.975 | SRR18125409 | SAMN26179459 |
| 75.A | Uzbekistan | 39.627 | 66.975 | SRR18125408 | SAMN26179460 |
| 76.A | Uzbekistan | 41.2995 | 69.2401 | SRR18125229 | SAMN26179461 |
| 77.A | Uzbekistan | 39.7681 | 64.4556 | SRR18125228 | SAMN26179462 |
| 79.B | Uzbekistan | 39.7681 | 64.4556 | SRR18125227 | SAMN26179463 |
| 7A | China | 47.1216 | 128.7382 | SRR18125226 | SAMN26179464 |
| 8.B | China | 47.1216 | 128.7382 | SRR18125225 | SAMN26179465 |
| 81.B | Afghanistan | 34.1769 | 61.7006 | SRR18125224 | SAMN26179466 |
| 82.B | Afghanistan | 34.1769 | 61.7006 | SRR18125222 | SAMN26179467 |
| 83.A | Afghanistan | 34.1769 | 61.7006 | SRR18125221 | SAMN26179468 |
| 84.A | Afghanistan | 34.1769 | 61.7006 | SRR18125220 | SAMN26179469 |
| 85.A | Afghanistan | 34.1769 | 61.7006 | SRR18125219 | SAMN26179470 |
| 86.A | Afghanistan | 36.6153 | 66.9293 | SRR18125218 | SAMN26179471 |
| 88.B | Afghanistan | NA | NA | SRR18125217 | SAMN26179472 |
| 89.B | Afghanistan | 33.9391 | 67.71 | SRR18125216 | SAMN26179473 |
| 90.B | Uzbekistan | NA | NA | SRR18125215 | SAMN26179474 |
| 91.A | Japan | 35.719 | 139.7456 | SRR18125214 | SAMN26179475 |
| 92.A | Japan | 35.719 | 139.7456 | SRR18125213 | SAMN26179476 |
| 93.B | Armenia | 40.0691 | 45.0382 | SRR18125211 | SAMN26179477 |
| 94.A | Iran | 35.102 | 59.1042 | SRR18125210 | SAMN26179478 |
| 95.A | Azerbaijan | 40.4093 | 49.8671 | SRR18125209 | SAMN26179479 |
| 96.A | Azerbaijan | 40.1431 | 47.5769 | SRR18125208 | SAMN26179480 |
| 97.A | Turkmenistan | 39.0041 | 63.5688 | SRR18125207 | SAMN26179481 |
| 98.B | Panama | 9.3593 | -79.8999 | SRR18125206 | SAMN26179482 |
| 99.A | Panama | 9.3593 | -79.8999 | SRR18125205 | SAMN26179483 |
| 99.B | Panama | 9.3593 | -79.8999 | SRR18125204 | SAMN26179484 |
| 9A | China | 47.1216 | 128.7382 | SRR18125203 | SAMN26179485 |
| M7.A | India | 31.5204 | 74.3587 | SRR18125202 | SAMN26179486 |

**Supplementary file 1b.** Outgroup *f*3 statistics among all possible combinations of genetic group pairs

| **Outgroup (C)** | **Source1**  **(A)** | **Source2**  **(B)** | ***f*3** | **Standard error** | **Z-score** | **Significant** |
| --- | --- | --- | --- | --- | --- | --- |
| *sublobata* | CA | EA | 0.232 | 0.005 | 42.66 | Yes |
| *sublobata* | CA | SA | 0.209 | 0.005 | 38.41 | Yes |
| *sublobata* | CA | SEA | 0.213 | 0.005 | 39.03 | Yes |
| *sublobata* | EA | SA | 0.209 | 0.005 | 38.79 | Yes |
| *sublobata* | EA | SEA | 0.218 | 0.005 | 40.75 | Yes |
| *sublobata* | SA | SEA | 0.211 | 0.005 | 39.41 | Yes |

Abbreviations: SA, South Asia; SEA, Southeast Asia; EA, East Asia and CA, Central Asia

(*f*3 statistics with Z-score > |3| are considered significant)

**Supplementary file 1c.** Admixture *f*3 statistics among all possible population trios

| **Target**  **(C)** | **Source1**  **(A)** | **Source2**  **(B)** | ***f*3** | **Standard error** | **Z-score** | **Significant** |
| --- | --- | --- | --- | --- | --- | --- |
| EA | SA | CA | 0.005 | 0.001 | 4.82 | Yes |
| EA | SEA | CA | -0.001 | 0.001 | -0.51 | No |
| EA | SEA | SA | 0.020 | 0.002 | 13.48 | Yes |
| SEA | CA | EA | 0.030 | 0.002 | 14.7 | Yes |
| SEA | SA | CA | 0.014 | 0.002 | 8.82 | Yes |
| SEA | SA | EA | 0.009 | 0.001 | 6.86 | Yes |
| SA | CA | EA | 0.032 | 0.002 | 16.66 | Yes |
| SA | CA | SEA | 0.011 | 0.001 | 9.02 | Yes |
| SA | EA | SEA | 0.017 | 0.001 | 12.91 | Yes |
| CA | EA | SA | 0.011 | 0.001 | 9.55 | Yes |
| CA | EA | SEA | 0.016 | 0.002 | 10.37 | Yes |
| CA | SEA | SA | 0.031 | 0.002 | 15.65 | Yes |

Abbreviations: SA, South Asia; SEA, Southeast Asia; EA, East Asia and CA, Central Asia

(*f*3 statistics with Z-score > |3| are considered significant, but only negative *f3* statistics denote the target population being admixed from source1 and source2.)

**Supplementary file 1d.** Mantel tests for isolation by distance of inferred genetic group (Q ≥ 0.5)

| **Group** | ***r*** | ***P*** |
| --- | --- | --- |
| SA | 0.4319 | 0.008* |
| SEA | 0.3312 | 0.041* |
| EA | 0.0461 | 0.052 |
| CA | 0.0070 | 0.435 |
| Southern | 0.2934 | 0.001* |
| Northern | 0.2777 | 0.001* |

Abbreviations: SA, South Asia; SEA, Southeast Asia; EA, East Asia; CA, Central Asia; *r*, Mantel correlation; significance level * *P* < 0.05

**Supplementary file 1e.** Description of bioclimatic variables used in ecological niche modelling

| **Bioclimatic variable** | **Variable** | **Unit** |
| --- | --- | --- |
| Bio1 | Annual mean temperature | ^o^C |
| Bio2 | Mean diurnal range (mean of monthly (max temp - min temp)) | ^o^C |
| Bio3 | Isothermality (Bio2/Bio7) (×100) | ^o^C |
| Bio4 | Temperature seasonality (standard deviation ×100) | ^o^C |
| Bio5 | Max temperature of warmest month | ^o^C |
| Bio6 | Min temperature of coldest month | ^o^C |
| Bio7 | Temperature annual range (Bio5-Bio6) | ^o^C |
| Bio8 | Mean temperature of wettest quarter | ^o^C |
| Bio9 | Mean temperature of driest quarter | ^o^C |
| Bio10 | Mean temperature of warmest quarter | ^o^C |
| Bio11 | Mean temperature of coldest quarter | ^o^C |
| Bio12 | Annual precipitation | mm |
| Bio13 | Precipitation of wettest month | mm |
| Bio14 | Precipitation of driest month | mm |
| Bio15 | Precipitation seasonality (coefficient of variation) | mm |
| Bio16 | Precipitation of wettest quarter | mm |
| Bio17 | Precipitation of driest quarter | mm |
| Bio18 | Precipitation of warmest quarter | mm |
| Bio19 | Precipitation of coldest quarter | mm |

**Supplementary file 1f.** Pearson’s correlation coefficient between pairs of bioclimatic variables (denoted in lower triangle)

**Supplementary file 1g.** Comparison of bioclimatic variables among the four genetic groups analysed with multivariate analysis of variance (MANOVA)

| **Predictor** | **Test statistic** | **Df** | **Observed value** | **F value** | **Num. Df** | **Den. Df** | ***P*** |
| --- | --- | --- | --- | --- | --- | --- | --- |
| Genetic group | Pillai | 3 | 1.790 | 44.215 | 24 | 717 | < 2e-16 |
|  | Wilks | 3 | 0.024 | 74.690 | 24 | 688 | < 2e-16 |
|  | Hotelling-Lawley | 3 | 12.716 | 124.870 | 24 | 707 | < 2e-16 |
|  | Roy | 3 | 10.777 | 321.960 | 8 | 239 | < 2e-16 |

Df = degree of freedom among groups; Num. Df = degrees of freedom of the model; Den. Df = degree of freedom of residual

**Supplementary file 1h.** Summary of analysis of variance (ANOVA) for bioclimatic variables

| **Bioclimatic variable** | **Df** | **Sum square** | **Mean square** | **F value** | ***P*** |
| --- | --- | --- | --- | --- | --- |
| Bio1 (Annual temperature) | 3 | 183.688 | 61.229 | 235.97 | <2.2e-16 |
| Bio2 (Mean diurnal temperature range) | 3 | 66.253 | 22.085 | 29.813 | <2.2e-16 |
| Bio3 (Isothermality) | 3 | 188.031 | 62.677 | 259.34 | <2.2e-16 |
| Bio8 (Mean temperature of wettest quarter) | 3 | 177.063 | 59.021 | 205.91 | <2.2e-16 |
| Bio12 (Annual precipitation) | 3 | 157.890 | 52.630 | 144.11 | <2.2e-16 |
| Bio14 (Precipitation of driest month) | 3 | 29.859 | 9.952 | 11.183 | <6.6e-07 |
| Bio15 (Precipitation seasonality) | 3 | 119.810 | 39.938 | 76.62 | <2.2e-16 |
| Bio19 (Precipitation of coldest quarter) | 3 | 44.212 | 14.737 | 17.732 | < 1.9e-10 |

Df = degree of freedom

**Supplementary file 1i.** Correlation between eight bioclimatic variables and climatic PC axes 1 to 4

| **Bioclimatic variable** | **PC1** | **PC2** | **PC3** | **PC4** |
| --- | --- | --- | --- | --- |
| Bio1 (Annual temperature) | **-0.445** | -0.107 | 0.390 | -0.056 |
| Bio2 (Mean diurnal temperature range) | 0.193 | **-0.564** | 0.062 | 0.253 |
| Bio3 (Isothermality) | **-0.430** | -0.082 | **0.492** | -0.184 |
| Bio8 (Mean temperature of wettest quarter) | **-0.486** | 0.042 | -0.287 | 0.049 |
| Bio12 (Annual precipitation) | **-0.401** | 0.366 | 0.066 | 0.202 |
| Bio14 (Precipitation of driest month) | 0.017 | **0.582** | -0.248 | 0.259 |
| Bio15 (Precipitation seasonality) | -0.323 | -0.365 | -0.292 | **0.667** |
| Bio19 (Precipitation of coldest quarter) | 0.279 | 0.235 | **0.607** | **0.587** |

(Correlation coefficients with absolute values higher than 0.4 are in bold.)

**Supplementary file 1j.** Comparison of summer growing season data including temperature and precipitation of May, July and September among the four genetic groups analysed with multivariate analysis of variance (MANOVA)

| **Predictor** | **Test statistic** | **Df** | **Observed value** | **F value** | **Num. Df** | **Den. Df** | ***P*** |
| --- | --- | --- | --- | --- | --- | --- | --- |
| Genetic group | Pillai | 3 | 1.915 | 70.907 | 18 | 723 | < 2e-16 |
|  | Wilks | 3 | 0.010 | 156.350 | 18 | 676 | < 2e-16 |
|  | Hotelling-Lawley | 3 | 19.873 | 262.400 | 18 | 713 | < 2e-16 |
|  | Roy | 3 | 15.400 | 618.570 | 6 | 241 | < 2e-16 |

Df = degree of freedom among groups; Num. Df = degrees of freedom of the model; Den. Df = degree of freedom of residual

**Supplementary file 1k.** ANOVA table for all evaluated field traits (phenology, reproduction and size) as well as drought-related traits

| **Trait** | **Garden** | **Model r^2** | **Group *F*** | **Group *P*** | **SEA^1^** | **SA^1^** | **CA^1^** | **Tukey^2^** |
| --- | --- | --- | --- | --- | --- | --- | --- | --- |
| **Phenology:** |  |  |  |  |  |  |  |  |
| Days to 50% flowering | Pakistan 2015 | 0.2388 | 7.2144 | 0.0019* | -0.3702 | 0.6296 | -0.3525 | B,A,B |
| Days to 50% flowering | Taiwan 1984 | 0.5266 | 25.5887 | <.0001* | 0.5723 | 0.3197 | -1.0685 | A,A,B |
| Days to 50% flowering | Taiwan 2018 | 0.4465 | 18.5544 | <.0001* | 0.0974 | 0.6569 | -0.9633 | A,A,B |
| **Reproduction:** |  |  |  |  |  |  |  |  |
| 100 seed weight, g | Pakistan 2015 | 0.4722 | 20.5761 | <.0001* | 0.9050 | -0.6397 | -0.2756 | A,B,B |
| Pod length, cm | Pakistan 2015 | 0.2621 | 8.1698 | 0.0009* | 0.6810 | -0.2605 | -0.4896 | A,B,B |
| Pod length, cm | Taiwan 1984 | 0.7173 | 58.3615 | <.0001* | 1.0524 | -0.1980 | -1.0232 | A,B,C |
| Pods per plant | Pakistan 2015 | 0.4471 | 18.5988 | <.0001* | -0.6441 | 0.8455 | -0.3062 | B,A,B |
| 1000 seed weight, g | Taiwan 1984 | 0.6248 | 38.3053 | <.0001* | 0.9979 | -0.8380 | -0.1340 | A,C,B |
| Seed yield per plant, g | Pakistan 2015 | 0.4666 | 20.1225 | <.0001* | -0.6210 | 0.8752 | -0.3712 | B,A,B |
| Seeds per pod | Pakistan 2015 | 0.1300 | 3.4372 | 0.0406* | -0.4875 | 0.2418 | 0.2806 | A,A,A |
| Seeds per pod | Taiwan 1984 | 0.1611 | 4.4168 | 0.0176* | 0.1413 | 0.3386 | -0.6107 | AB,A,B |
| **Plant size:** |  |  |  |  |  |  |  |  |
| Petiole length, cm | Pakistan 2015 | 0.2943 | 9.5907 | 0.0003* | 0.5435 | 0.0878 | -0.7798 | A,A,B |
| Plant height, cm | Pakistan 2015 | 0.0001 | 0.0024 | 0.9976 | 0.0075 | 0.0055 | -0.0158 | A,A,A |
| Plant height at flowering, cm | Taiwan 1984 | 0.3981 | 15.2115 | <.0001* | 0.4811 | 0.3024 | -0.9705 | A,A,B |
| Plant height at maturity, cm | Taiwan 1984 | 0.5472 | 27.8000 | <.0001* | 0.3480 | 0.5605 | -1.1362 | A,A,B |
| Primary leaf length, cm | Taiwan 1984 | 0.5454 | 27.5930 | <.0001* | 0.9813 | -0.4212 | -0.6253 | A,B,B |
| Primary leaf width, cm | Taiwan 1984 | 0.6053 | 35.2773 | <.0001* | 1.0244 | -0.6010 | -0.4313 | A,B,B |
| Terminal leaflet length, cm | Pakistan 2015 | 0.2186 | 6.4340 | 0.0034* | 0.3062 | 0.2643 | -0.7167 | A,A,B |
| Terminal leaflet width, cm | Pakistan 2015 | 0.1680 | 4.6458 | 0.0145* | 0.4361 | 0.0387 | -0.5734 | A,AB,B |
| **Drought (PEG6000):** |  |  |  |  |  |  |  |  |
| Shoot dry weight (SDW), mg | NTU 2021 | 0.5998 | 36.7246 | <.0001* | 1.0508 | -0.5607 | -0.5483 | A,B,B |
| Root dry weight (RDW), mg | NTU 2021 | 0.5964 | 36.2048 | <.0001* | 1.0299 | -0.6933 | -0.3336 | A,B,B |
| Total dry weight (TDW), mg | NTU 2021 | 0.5934 | 35.7555 | <.0001* | 1.0448 | -0.5883 | -0.5028 | A,B,B |
| Root:Shoot ratio dry weight (RSRDW) | NTU 2021 | 0.3261 | 11.8577 | <.0001* | -0.2316 | -0.4342 | 0.9112 | B,B,A |
| **Drought (Control):** |  |  |  |  |  |  |  |  |
| Shoot dry weight (SDW), mg | NTU 2021 | 0.5779 | 33.5453 | <.0001* | 1.0275 | -0.4709 | -0.6484 | A,B,B |
| Root dry weight (RDW), mg | NTU 2021 | 0.5205 | 26.5962 | <.0001* | 0.9716 | -0.6077 | -0.3811 | A,B,B |
| Total dry weight (TDW), mg | NTU 2021 | 0.5638 | 31.6722 | <.0001* | 1.0195 | -0.5374 | -0.5430 | A,B,B |
| Root:Shoot ratio dry weight (RSRDW) | NTU 2021 | 0.1753 | 5.2083 | 0.0089* | -0.3036 | -0.2004 | 0.6773 | B,B,A |

*Significant at *P* < 0.05; 1: least-square means of each group after inverse normal transformation of raw data; 2: levels not connected by same letter are significantly different.

**Supplementary file 1l.** Mean of eight bioclimatic variables of the genetic groups

| **Bioclimatic variable** | **Northeast Asia** | **Northwest Asia** | **Southeast Asia** | **South Asia** | **Central Asia** |
| --- | --- | --- | --- | --- | --- |
|  | **(N = 37)** | **(N = 45)** | **(N = 45)** | **(N = 49)** | **(N = 72)** |
|  | **Mean ± SD** | **Mean ± SD** | **Mean ± SD** | **Mean ± SD** | **Mean ± SD** |
| **Bio1** | 62.49 ± 55.39 | 117.58 ± 54.30 | 256.47 ± 19.29 | 255.57 ± 14.80 | 128.72 ± 41.10 |
| **Bio2** | 115.22 ± 15.44 | 124.38 ± 20.14 | 102.02 ± 16.11 | 127.24 ± 16.12 | 130.08 ± 13.89 |
| **Bio3** | 25.14 ± 2.04 | 31.02 ± 4.47 | 51.58 ± 7.19 | 42.8 ± 4.58 | 32.4 ± 3.52 |
| **Bio8** | 207.86 ± 31.97 | 107.89 ± 67.90 | 271.47 ± 11.68 | 283.92 ± 23.02 | 92.04 ± 42.23 |
| **Bio12** | 821.59 ± 299.98 | 301.24 ± 201.00 | 1477.69 ± 380.18 | 750.39 ± 329.19 | 285.67 ± 145.24 |
| **Bio14** | 12.14 ± 11.72 | 5.18 ± 8.99 | 6.33 ± 4.34 | 2.47 ± 3.44 | 2.51 ± 4.98 |
| **Bio15** | 95.35 ± 24.52 | 65.4 ± 20.35 | 84.78 ± 7.25 | 124.47 ± 23.48 | 70.92 ± 12.92 |
| **Bio19** | 42.51 ± 38.23 | 88.33 ± 67.16 | 48.02 ± 24.91 | 34 ± 33.99 | 92.15 ± 45.19 |
